# Seasonal Effect on Developmental Competence, Oxidative Status and Tubulin Assessment of Prepubertal Ovine Oocyte

**DOI:** 10.3390/ani11071886

**Published:** 2021-06-24

**Authors:** Elisa Serra, Sergio Domenico Gadau, Giovanni Giuseppe Leoni, Salvatore Naitana, Sara Succu

**Affiliations:** Department of Veterinary Medicine, University of Sassari, Via Vienna 2, 07100 Sassari, Italy; eliserra@uniss.it (E.S.); gioleoni@uniss.it (G.G.L.); snaitana@uniss.it (S.N.); succus@uniss.it (S.S.)

**Keywords:** oocyte, lamb, season, oxidative status, developmental competence

## Abstract

**Simple Summary:**

Oocytes obtained from the ovaries of slaughtered prepubertal ewes can be incorporated into an in vitro embryo production system. The collection of this material is possible at two different times of the year, spring and autumn. The first period is linked to the natural reproductive cycle of the sheep. The second, on the other hand, is linked to the manipulation of the reproductive seasonality which allows the oestrus cycle to be controlled artificially. The analysis highlighted that the collection season influences oocytes quality from prepubertal donors in terms of improved energetic and oxidative status, microtubular organization, and developmental competence in oocytes recovered in spring. Data obtained underline that oocytes seem to be genetically and evolutionarily programmed to give their best in spring, this being the most favorable period for newborns.

**Abstract:**

The reproductive seasonality of domestic animals is often manipulated in order to have more reproductive periods for commercial purposes related to the production of milk and meat. It is scientifically proven that such an alteration of the reproductive activity in sheep entails a deterioration in oocyte quality, leading to an inability to generate embryos. Since oocytes obtained from prepubertal ewes can be incorporated into an in vitro embryo production system and considering that their quality is crucial to the success of in vitro procedures, the aim of this work was to investigate the effect of seasons on the quality of prepubertal ovine oocytes collected in autumn and spring. Ovaries were collected from a local slaughterhouse from 30–40-day-old suckling lambs during both seasons. Following 24 h of in vitro maturation, oocytes developmental competence, reactive oxygen species (ROS) intracellular levels, and mitochondrial activity were evaluated, and a tubulin assessment was performed. The results on embryo production, as a percentage of first divisions and number of blastocysts obtained, were significantly higher in oocytes collected in the spring. Mitochondrial activity in oocytes was higher, and ROS production significantly lower, in spring than in autumn. Tubulin PTMs (tyrosinated and acetylated α-tubulin) showed a higher immunoreactivity in oocytes collected in spring compared with autumn sampling. Our data showed that seasons may affect the developmental competence, energetic status, and tubulin assessment of oocytes recovered from prepubertal ewes. Therefore, special care should be taken when choosing the period of the year for prepuberal ovine oocytes collection aimed at in vitro embryo reproduction programs.

## 1. Introduction

According to geneticists, accelerating the reproductive rate of a breed or a single animal represents the starting point to achieve a good selection program. The application of such selection programs shows limitations in several animal species, depending on the reproductive seasonality; a sort of ancestral inheritance resulting from the adaptation achieved by animals to survive during the natural selection process. Indeed, seasonal breeding is a survival strategy adopted by many wild animals to ensure that their progeny are born at the most favorable time of year, with the most appropriate environmental conditions resulting in higher chances of offspring survival [1]. The reproductive seasonality also characterizes several domestic species such as sheep. Reproductive activity, controlled by photoperiod and regulated by the melatonin inhibiting Gonadotropin-Inhibitory Hormone (GnIH) [2] and stimulating Kisspeptin-Neurokinin-Dynorphin (KNDy) neurons of the arcuate nuclei to increase Gonadotropin Releasing Hormone (GnRH) pulse patterns [3], represents the successful adaptation of these animals to natural mating for one season per year. Moreover, in Sardinia, an extension of the sheep breeding season has been obtained through distributing lambing along the year to ensure milk production for several additional months as well as to satisfy the meat market demand. This is achieved using the out-of-breeding mating strategy, which consists of interrupting sexual promiscuity by the use of the male effect and exogenous hormones for oestrus cycle control [4]. In this way the lambing calendar is modified in order to obtain 30–40-day-old lambs (weighing 8–10 kg) during the Easter and Christmas celebrations. However, and despite their wide and frequent use, these strategies may result in decreased fertility [5]. In in vitro embryo production programs (IVP) several authors have highlighted a periodic reduction in embryo yield in both cattle and buffalo [6]. Also, a decrease in oocyte quality during the non-breeding season has been shown in cats and monkeys [7,8]. As for sheep, it has been reported that seasonality influences the number of in vitro embryo production [9].

The influence of oocyte competence on embryo developmental potential is now well recognized [10]. Oocyte competence is the ability of oocytes to resume meiosis, to cleave following fertilization, and to develop to the blastocyst stage. It represents the quality marker commonly used by most laboratories [11]. Among various cellular mechanisms involved in oocyte competence, mitochondrial activity can be included [12,13,14,15] (as it is essential for energy production required for fertilization and embryo development), concentration of reactive oxygen species (ROS) [10,14,16,17], and for microtubular cytoskeleton development [18,19]. The microtubules (MTs) and their post-translational modifications (PTMs) may have an important role during the maturation and the fertilization of the oocyte due to their involvement in the meiotic spindle assembly and the subsequent segregation, alignment, and movement of chromosomes [20]. In our previous work [21], we found that the most abundant tubulin PTMs in the meiotic spindle of ovine oocytes are tyrosination and acetylation. PTMs, as well as acetylation, characterizes more stable MTs, while tyrosination is more abundant on the more dynamic ones [22]. Therefore, the tubulin PTMs composition in the MTs of the meiotic spindle may be a crucial factor involved in oocyte competence [21,23]. Since oocytes obtained from ovaries of slaughtered prepubertal ewes can be incorporated into an in vitro embryo production system [24], and considering that their quality is crucial to the success of in vitro procedures, the present work aimed to describe a possible relationship between collection season and quality in ovine prepubertal oocytes. Therefore, in the present study the quality of oocytes collected during autumn (November–December) and spring (March–April) was investigated through an evaluation of developmental competence, mitochondrial activity, ROS intracellular levels, and a tubulin assessment. 

## 2. Materials and Methods

All chemicals in this study were purchased from Sigma Chemical CO (St. Louis, MO, USA) unless stated otherwise. 

### 2.1. Oocyte Collection and In Vitro Maturation

A total of 2495 in vitro matured oocytes were used: 1054 oocytes collected in autumn (November–December) and 1441 oocytes collected in spring (March–April) of the same year. In total, 332 prepubertal Sarda sheep were slaughtered and 7.5 ± 1.56 oocytes per animal were obtained for this study. The collection of oocytes and their subsequent maturation was carried out as previously described [21,23]. Briefly, ovaries collection from prepubertal ewes slaughtered for commercial purpose (30–40 days old; weight of 8–10 Kg; not weaned) was performed over 1–2 h. Cumulus oocyte complexes (COCs) were selected according to morphological standard (4–10 layers of granulosa cells, uniform cytoplasm homogenous distribution of lipid droplets) and matured in vitro in TCM 199 with supplements heat-treated estrous sheep serum (10%), Follicle-Stimulating Hormone (FSH; 1 IU/mL), Luteinizing Hormone (LH; 1 IU/mL), cysteamine (100 mM), and pyruvate (8 mg/mL). Thirty-five COCs were transferred to 600 µL of maturation medium in four-well Petri dishes (Nunclon, Nalge Nunc International, Denmark) and cultured in standard conditions (22 h in 5% CO_2_ in air at 39 °C). Following in vitro maturation, the COCs were mechanically denuded of cumulus cells and only those at metaphase II (MII) were selected and randomly assigned to the analyses.

### 2.2. Determination of Oocytes Developmental Competence

In vitro matured oocytes were fertilized with frozen/thawed spermatozoa from the same ejaculate of a single ram. The in vitro fertilization procedures (IVF) were conducted as previously described in standard conditions [14]. Briefly, cryopreserved semen was obtained from rams with proven fertility. The fertilization medium was composed of synthetic oviductal fluid (SOF) supplemented with 2% heat-treated estrous sheep serum, 10 μg/mL heparin, and 1 μg/mL hypoutarine. The motile and capacitated sperm fraction was selected by swim-up after incubation for 15 min at 39 °C and in 5% CO_2_, 5% O_2_, and 90% N_2_ atmosphere. Swim-up-derived spermatozoa were co-incubated with in vitro matured oocytes at 39 °C and in 5% CO_2_, 5% O_2_, and 90% N_2_ in a maximum minimum atmosphere. After 22 h of fertilization (hpf), the presumptive zygotes were transferred in a SOF medium with bovine serum albumin (BSA 4 mg/mL) and essential and non-essential amino acids at oviductal concentration [25] and kept at 39°C, 5% O_2_, 5% CO_2_ (i.e., the maximum humidified atmosphere). The number of embryos showing the first mitotic division at 22, 26, and 32 hpf was recorded. Newly formed expanded blastocysts were recorded every day starting from the sixth until the ninth day post-fertilization (dpf).

### 2.3. Evaluation of Mitochondrial Activity and Reactive Oxygen Species (ROS) Intracellular Level

In vitro matured lamb oocytes were subjected to a simultaneous triple staining. First, oocytes were incubated in Phosphate Buffered Saline supplemented with 20% Fetal Calf Serum (PBS/20%FCS) for 30 min at 38.5 °C with Mito-Tracker Red CM-H_2_XRos (500 nM; MT-Red, Molecular Probes, Inc., Eugene, OR, USA), a mitochondrial-specific fluorescent and cell-permeant probe. Subsequently, after being washed three times in PBS/0.1%PVA, the oocytes were incubated for 20 min in the same medium with 20, 70-dichlorodihydrofluorescein diacetate (5 mM; H_2_DCF-DA, Molecular Probe, Eugene, OR, USA), a reactive oxygen species (ROS) active molecular probe. Finally, the oocytes were counterstained with Hoechst 33342 in order to stain the chromosomes. After exposure to probes, the oocytes were washed three times in fresh PBS/0.1%PVA and fixed in 2.5% glutaraldehyde/PBS for at least 15 min. After fixation, the oocytes were prepared for the evaluation by using a confocal microscopy (Leica TCS SP5 CLSM with Leica LAS lite 170 Image software, Wetzlar, Germany). For mitochondrial evaluation, samples were observed with a multiphoton laser to detect MitoTracker Red CM-H_2_XRos (ex: 579 nm; em: 599 nm). An argon ions laser ray at 488 nm and the B-2 A filter (495 nm exposure and 519 nm emission) were used to point out the DCF [14].

### 2.4. Tubulin Immunofluorescence and Confocal Microscope Evaluation

In order to evaluate tyrosinated α-tubulin (Tyr-T) and acetylated α-tubulin (Ac-T) by immunofluorescence, IVM oocytes were fixed in PBS/4% Paraformaldehyde solution and processed following the methodology illustrated previously [21,23,26]. In brief, the oocytes were processed through serial incubations (1 h per antibody at 37 °C) with the following primary antibodies: anti-Tyr-T (monoclonal, clone TUB-1A2, 1:1000) and anti-Ac-T (monoclonal, clone 6-11B-1, 1:1000). After rinsing with PBS/2%FCS, the cells were incubated with secondary anti-mouse fluorescein isothiocyanate-conjugated antibodies (FITC-AlexaFluor 488, Thermo Fisher Scientific, Waltham, MA, USA), then washed three times, counterstained with Hoechst 33342, and mounted on a slide for immunostaining evaluation. The analysis of immunolabeled sections were performed with a confocal laser scanning microscope from Leica (TCS SP5 DMI 6000CS, Leica Microsystems GmbH, Wetzlar, Germany), equipped with Ar/He/Ne lasers, using a 40/60 X oil objective following the procedures standardized by Gadau [26,27]. Briefly, the sections were analyzed by sequential excitation of the two fluorochromes used (Hoechst 33342 and FITC 488), and parameters related to fluorescence intensity were maintained at constant values during all image acquisitions. Quantitative analysis of fluorescence intensity was performed using the Leica LAS AF Lite image analysis software package (Leica Microsystems GmbH, Wetzlar, Germany).

### 2.5. Statistical Analysis

For the statistical analysis, the variables used were mitochondrial activity, ROS intracellular level, Tyr-T, and Ac-T fluorescence intensity. For each variable, the difference between spring and autumn was evaluated by ANOVA one-way Analysis of Variance after analysis for homogeneity of variance by Levene’s test. The normal distribution of data was investigated by the Kolmogorov–Smirnov normality test, as most of the data were not normally distributed and data were log-transformed. All results were expressed as a mean ± mean standard error (S.E.M.). Differences in developmental competence of prepubertal ovine oocyte collected during spring and autumn after in vitro maturation were analyzed using the chi square test (χ^2^). Statistical analyses were performed using the statistical software Minitab^®^ 17.1 (2017 Minitab Inc., Coventry, UK) and a probability of *p* ≤ 0.05 was considered the minimum level of significance.

## 3. Results

### 3.1. Evaluation of Oocyte Developmental Competence

Data regarding the oocyte developmental competence are shown in Table 1. The total cleavage rate was significantly lower in oocytes collected in autumn compared with oocytes collected in spring (74.4 % vs. 80.1%, respectively; *p* < 0.01). No differences were observed in the cleavage of inseminated oocytes collected in spring and autumn at the different interval times post-fertilization (22–26–32 h).

Data on blastocyst production confirmed the cleavage data, with a lower rate of blastocysts derived from the autumn group compared with those derived from the spring group (12.5% vs. 19.8% respectively; *p* < 0.01). The distribution of blastocysts according to the day post-fertilization (dpf) culture in which they appeared, showed a delay of development in the autumn group. In the spring group, in fact, blastocyst production on the seventh dpf was significantly higher (46.5%) compared to autumn (13.8%; *p* < 0.01). After eight days of culture, the blastocysts rate was higher in the autumn group compared with the spring group (75.9% vs. 44.6% respectively; *p* < 0.01). No differences in blastocyst production were registered between the two groups after the ninth dpf.

### 3.2. Evaluation of Mitochondrial Activity and ROS Intracellular Level

**Figure 1:**
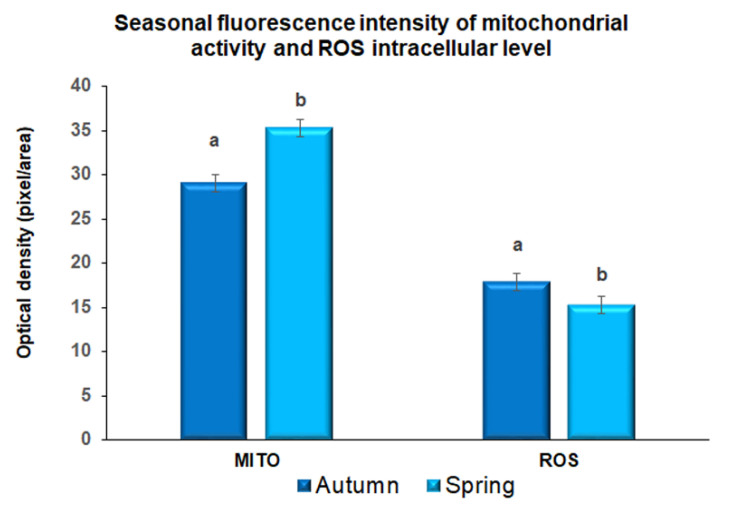
Quantification of fluorescence intensity after incubation with Mitotracker-Red stain (Mt-Red) and H_2_DCF-DA probe (reactive oxygen species, ROS) in metaphase II prepubertal ovine oocytes collected during autumn and spring. Values are expressed as arbitrary units (Mean ± mean standard error). Different lower-case letters indicate statistical difference into mitochondrial fluorescence intensity (*p* < 0.05 ANOVA). Different lower-case letters indicate statistical difference into DCHFDA fluorescence intensity (*p* < 0.05 ANOVA).

Fluorescence intensity data (measured as relative intensity (rfu), after incubation with the Mitotracker-Red probe and ROS sensitive fluorescent staining in five replicates, showed statistical differences between the two experimental groups. Mitochondrial activity was significantly higher (*p* < 0.05) in oocytes collected in spring compared with those collected in autumn (35.31 ± 14.17 vs. 29.07 ± 13.69 rfu respectively) while, on the contrary, ROS intracellular levels were significantly lower (*p* < 0.05) in oocytes collected in spring compared with oocytes collected in autumn (15.31 ± 9.29 vs. 17.87 ± 6.78 respectively; Figure 1).

### 3.3. Evaluation of Tubulin Assessment

**Figure 2:**
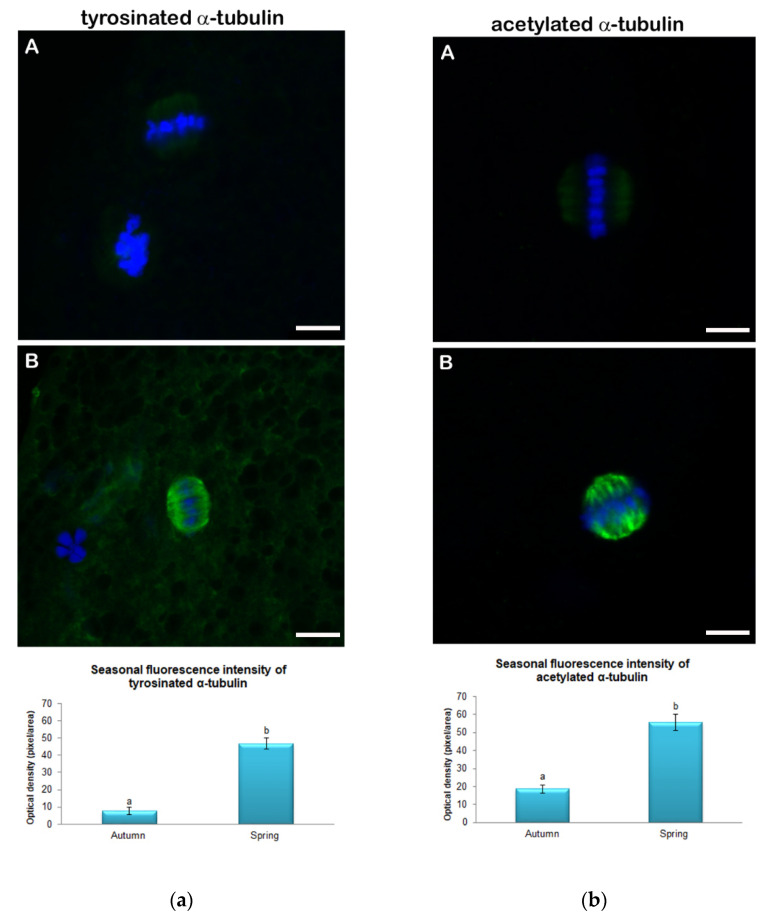
Quantification of fluorescence intensity of tyrosinated (**a**) and acetylated (**b**) α-tubulin in metaphase II prepubertal ovine oocytes collected during Autumn (**A**) and Spring (**B**). Values are expressed as arbitrary units (Mean ± mean standard error). Different lower-case letters indicate statistical difference in tyrosinated α-tubulin fluorescence intensity in autumn and spring (*p* < 0.01 ANOVA).

**Figure 3:**
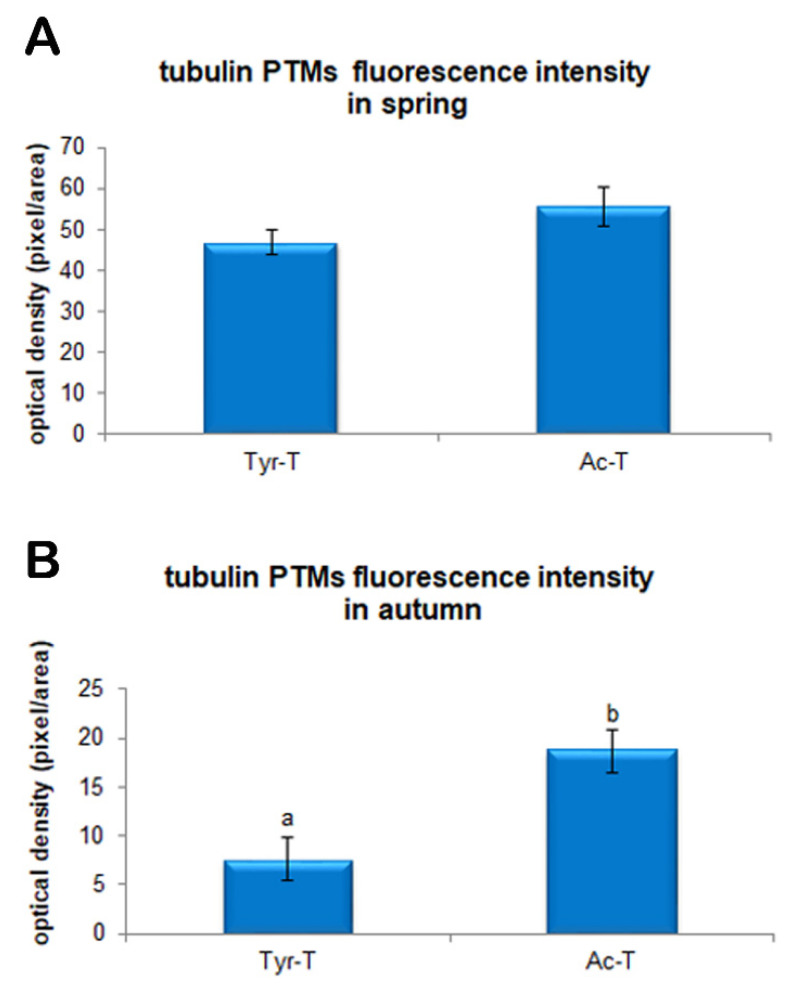
Quantification of fluorescence intensity for tyrosinated and acetylated α-tubulin in MII prepubertal ovine oocyte collected in autumn (**A**) and spring (**B**). Values are expressed as arbitrary units (Mean ± mean standard error). Different lower-case letters indicate statistical difference between tyrosinated and acetylated α-tubulin fluorescence intensity in autumn (**A**) and spring (**B**) metaphase II prepubertal ovine oocytes (*p* < 0.01 ANOVA).

Immunofluorescence for tyrosinated and acetylated α-tubulin, evaluated in five replicates displayed a clearly visible signal emission in the meiotic spindle of both spring and autumn prepubertal ovine oocytes. The signal intensity of both tyrosinated and acetylated α-tubulin was significantly higher (*p* < 0.01) in oocytes collected in spring than in oocytes collected in autumn. As shown in Figure 2a the levels of tyrosinated α-tubulin were 55.6 ± 4.6 vs. 18.7 ± 2.2 rfu in oocytes collected in spring and autumn, respectively. Instead, levels of acetylated α-tubulin, displayed in Figure 2b, were 46.8 ± 3.1 vs. 7.6 ± 2.2 rfu in oocytes collected in spring and autumn, respectively. Moreover, considering the fluorescence intensity of the two diverse tubulin PTMs within each experimental group, in the spring group no differences were shown between tyrosinated and acetylated α-tubulin fluorescence intensity (47.8 ± 3.1 vs. 55.6 ± 4.6 rfu in spring and autumn oocytes, respectively; Figure 3A). On the contrary, in the autumn group the fluorescence intensity of acetylated α-tubulin was significantly higher compared with tyrosinated α-tubulin (18.7 ± 2.2 vs. 7.6 ± 2.2 rfu in spring and autumn groups, respectively; *p* < 0.01; Figure 3B).

## 4. Discussion

The aim of the present study was to determine the effect of seasons on the oocyte quality of ewe lambs collected at the slaughterhouse during two different periods of the year (autumn and spring). Considering the pivotal role of oocytes in female reproduction, different authors have studied the role of seasonal variations in the developmental capacity of the oocyte and natural fertility in different seasonal breeder species such as sheep [28], goats [29], horses [30,31], and buffalo [5]. In sheep, the positive impact of the breeding season on oocyte quality and reproductive performance has been related to the higher number of recovered oocytes [32,33], higher cleavage rate [34], and higher blastocyst production [9]. Instead, the efficiency of ovum recovery procedures applied in the non-breeding season is affected in terms of elevated percentage of unfertilized oocytes and poor embryo viability, as well as a reduced cleavage rate after IVF of ovulated oocyte [35]. In adults, this is explained by physiological and hormonal changes and characteristics of the reproductive season that induce optimal follicular development and, hence, an increased oocyte quality [36,37]. In adult sheep, the higher cleavage [34] and blastocyst rate [9] of oocytes collected during the breeding season (rather than in anoestrous) is relative to the higher developmental competence of oocytes recovered during a decreasing photoperiod, when short days stimulate sexual activity.

With respect to oocytes from prepubertal animals, the effect of season still remains to be explored, However, our data has shown that in our geographical conditions the season may affect the quality of ovine oocytes from young donors. In prepubertal goats, it has been shown that the season affects the fatty acid composition in follicular fluid and, consequently, oocyte competence [38]. In this study, it has been observed that the lowest blastocyst production after IVF and parthenogenetic activation procedures are in autumn compared to winter and spring seasons, correlated with a lower n6: n3 PUFA ratio obtained in winter than in autumn. Furthermore, other authors have reported a higher cleavage rate in winter compared with autumn using oocytes from four to eight week-old lambs [39]. Our data are quite in line with results reported by these authors. The worst performances were in fact also recorded by our research group during autumn, even if compared only to spring due to the lack of slaughtered animals in winter for commercial reasons.

Despite the fact that oocytes collected during the two seasons reached metaphase II (Table 1) at the same rate, autumn oocytes showed a lower developmental competence compared to the spring group, meaning that the ability of the oocytes to reach meiotic maturation does not necessarily confirm the achievement of full developmental competence [40,41]. Indeed, lower cleavage and blastocysts rates were recorded in the autumn group and the lower developmental capacity of these oocytes was highlighted also in terms of the quality of produced embryos. The day of blastocyst appearance has been related to the embryo developmental potential [42]. In sheep, it has been demonstrated that earlier produced blastocysts show a higher viability and hatching rate after vitrification and warming. Thus, the days of blastocyst production reflect the quality of obtained embryos [43]. Early obtained blastocysts (seventh dpf) are numerically more elevated in the spring group, while in the autumn group the greatest number of blastocysts is obtained one day later with a delay of cell cycles during embryo culture [43]. It follows that reduced cleavage and blastocyst rates, and a subsequently reduced quality of produced embryos observed in the autumn group, could be related to a low quality of oocytes collected during this season compared with oocytes collected in spring.

Given that, as far as we know, the prepubertal endocrine environment is profoundly different than in adults due to the hypothalamic-pituitary-gonadal axis quiescent and minimal levels of sex steroids [44], we can speculate that season can induce local autocrine and paracrine changes, and may regulate follicular environment and, thus, oocyte quality in these young animals. Considering that the meiotic competence observed was similar in oocytes collected in spring and autumn, we can hypothesize that cytoplasmic competence is influenced by seasonal change. Indeed, an insufficient prepubertal oocyte capacitation during follicular growth induces reduced embryo quality and a failure of embryonic development [45,46]. In fact, the low quality of prepubertal oocyte cytoplasm has already been associated with an altered cytoplasmic molecular environment [12,47,48]. This finding was supported by our data on cytoplasmic mitochondrial activity and ROS intracellular levels. The regulation of intracellular redox potential in the oocyte is a crucial determinant of fertility and embryo development [12,14]. The presence of ROS within the cytoplasm of the oocyte is physiological, closely related to the oxidative metabolism, even though their effects are dose dependent. At low levels, ROS regulate specific cellular functions [49] while at high levels they produce oxidative stress on the cell resulting in molecular damage to DNA, proteins, and lipids [50]. Oxidative injury may induce the release of cytochrome c and other apoptogenic factors from mitochondria which eventually activate programmed cell death [51]. Oxidative stress has also been associated with impaired early development and fragmented embryos. In fact, the alteration of intracellular redox potential is associated with a reduction of mitochondrial activity and, as a consequence, negatively affects the bioenergetics status [50] decreasing ATP levels, compromising the progression of oocyte maturation and the development of preimplantation embryos [52,53]. Since mitochondria assume a vital role in the metabolism of energy-containing compounds in the ooplasm in order to provide ATP for fertilization and pre-implantation embryonic development, their role in ATP production is essential for oocyte quality [12]. In sheep oocytes, we demonstrated that high levels of mitochondrial activity and low ROS intracytoplasmic concentrations were related to greater developmental competence [14,43]. The present data evidenced a higher mitochondrial activity and a lower ROS level in spring oocytes compared to autumn ones, evidencing the improved quality of oocytes collected during spring season.

Even data resulting from the analysis of the post-translational modifications of tubulin would seem to confirm this hypothesis. The importance of the microtubular network in the oocyte is well known, as it is involved in the formation of the meiotic spindle, chromosome segregation, fertilization, and embryonic development [54,55]. The data resulting from the fluorescence analysis of tyrosinated and acetylated α-tubulin revealed a different intensity pattern between the two seasonal periods examined, with a clear balance between the two PTMs in oocytes harvested during spring which could be considered a marker of a “highly competent system” [21]. This balance is the basis of the normal functionality of a meiotic spindle, a structure that must be equipped with dynamism and stability at the same time. It is well known that tyrosination is a typical modification of dynamic and unstable microtubules [56,57,58]. The latter move in the cell cytoplasm in search of their target, which in the case of the meiotic spindle are chromosomes [59,60]. After the search and capture phase, stabilization of microtubules through acetylation appears to be at the basis of normal chromosome alignment, and appears to consolidate once the microtubule contacts the kinetochore [61,62]. Furthermore, tubulin acetylation confers resistance to traction forces in those curved structures such as the meiotic spindle [63]. In the evaluation of the immunopositivity level of tubulin PTMs in autumn lamb oocytes, we can speculate that the balance between the stability and dynamism of the microtubule is lacking, since oocytes displayed higher levels of acetylated than tyrosinated tubulin. A higher immunopositivity of acetylated tubulin due to a predominant acetylation on the microtubules may lead to morphological alterations of the meiotic spindle and to alignment defects [21,64]. This imbalance in the composition of tubulin PTMs may be an additional factor causing poor developmental competence of lamb oocytes collected in the autumn period, as it would threat the intrinsic “dynamic stability” of the microtubules [65].

In addition, maternal stress and nutrition during pregnancy might influence the environment in the uterus and the development of fetal ovaries and/or oocyte development of the offspring. The semi-extensive farming system of dairy sheep in Mediterranean countries, as well as in Sardinia, is based mainly on pastures whose quantitative and qualitative availability is influenced by environmental conditions and by season [66,67]. In our latitude, in early spring from March to April, the highest quality of pasture can be found with a subsequent decrease in forage quality during the following months due to the fact that plants mature from the vegetative to the reproductive stage [68]. Several authors reported that seasonal, and consequently nutritional differences, in pastures influences sheep milk composition, milk yield [69,70,71,72], and also affects the carcass and meat quality of suckling lambs [73]. It appears that lambs born and slaughtered in spring have a high quantity and quality of milk available to them compared with those born and slaughtered in autumn. Inevitably, such more satisfactory energetic conditions positively affect the ovarian environment. These could be some of the reasons to explain why lambs born in autumn show a different ovarian status with less oocyte competence compared to oocytes collected in the spring season. Further studies are needed to elucidate mechanisms involved in the acquisition of prepubertal ovine oocyte quality in relation to the season.

## 5. Conclusions

Our data show that season may affect developmental competence, energetic status, and tubulin assessment of oocytes recovered from prepubertal ewes. Therefore, careful attention should be paid in choosing the period of the year for prepuberal ovine oocytes collection for in vitro embryo reproduction programs.

## Figures and Tables

**Table 1:** Developmental competence of prepubertal ovine oocytes collected during spring and autumn.

Season	No. of Collected Oocytes	In Vitro Matured and Inseminated Oocytes (%)	Cleavage (%)			Blastocyst (%)				
					Tot *	7 dpf	8 dpf	9 dpf	Tot **	Tot ***
Autumn	898	624	351 (75.6)	113 (24.4)	464 ^a^ (74.4)	8 ^a^ (13.8)	44 ^a^ (75.9)	6 (10.3)	58 ^a^ (12.5)	58 ^a^
		(69.5)								(6.5)
Spring	1373	1005	575 (71.4)	230 (28.6)	805 ^b^ (80.1)	74 ^b^ (46.5)	71 ^b^ (44.6)	14 (8.8)	159 ^b^ (19.8)	159 ^b^ (11.6)
		(73.2)								

* The percentage of total cleavage is calculated on the number of fertilized oocytes, while the cleavage during the two interval post-fertilization is calculated on total cleaved oocytes. ** The percentage of total blastocysts is calculated on the number of total cleaved oocytes, while distribution across the post-fertilization days is calculated as rate on the total blastocysts; *** The percentage of total blastocysts is calculated on the number of collected oocytes. hpf, hours post-fertilization; dpf, days post-fertilization. Within the same column, different letters indicate statistical differences, *p* < 0.01 (Chi-square test).

## Data Availability

Data presented in this study are available on request from the corresponding author.
